# Acute Stress Assessment From Excess Cortisol Secretion: Fundamentals and Perspectives

**DOI:** 10.3389/fendo.2019.00749

**Published:** 2019-11-05

**Authors:** Patrice Boucher, Pierrich Plusquellec

**Affiliations:** ^1^Software and Information Technology Engineering Department, École de Technologie Supérieure, Montreal, QC, Canada; ^2^Centre d'Études sur le Stress Humain, École de PsychoÉducation, Centre de Recherche de l'Institut Universitaire en Santé Mentale de Montréal, Université de Montréal, Montreal, QC, Canada

**Keywords:** stress, allostasis, cortisol, psychophysiology, measurements, regulation, control theory

## Abstract

Our paper aims to redefine the concept of stress in the context of maintaining allostasis; the term has been reserved for situations that concomitantly involve established physiological and psychological stress components. In particular, we analyze how novelty, unpredictability, threat to the ego, and low sense of control challenge allostasis. The concept of stress is then related to *a state of difficulty in maintaining allostasis*, rather than referring to the overall body response to the situation. This state of difficulty may be observed either in planning the strategy to deal with the situation, evaluating consequent target trajectories for the actuators, the catabolic mediators and the activators, or regulation of the biological systems through these trajectories. Catabolic mediator excesses are proposed as scaling the level of difficulty in maintaining allostasis. The *excess proportion of cortisol load* (*EPCL*) is consequently proposed to scale the stress level. A first proof-of-concept of this indicator is realized using the *Physiostress* dataset, by asserting that it is, as predicted from its theoretical basis, more in phase with the stress level expected from the nature of the task and participant-reported stress compared to common indicators based on the cortisol response magnitude itself.

## Introduction

The effects of psychological stress on the cortisol response were first studied from the introduction of the human stress concept by (1), then by many of his contemporary researchers [e.g., (2)]. Stress was first defined by Selye as a “*non-specific response of the body to any demand for change*.” This broad-spectrum definition was also reformulated by some authors as: “*a state of disharmony, or threatened homeostasis*,” (3) and when talking about the stress response, as “*a general alarm in a homeostatic system, producing general and unspecific neurophysiological activation from one level of arousal to more arousal*” (4). More recently, stress was defined as “*a real or interpreted threat to an individual physiological and psychological integrity that results in adaptive biological and behavioral responses*” (5, 6). Finally, Dickerson and Kemeny (7) reviewed 208 studies on stress to specifically demonstrate that “*uncontrollable threats to the social-self elicit robust and reliable cortisol responses*.” Consistently, Koolhaas et al. (8) restricted the terms *stress* and *stressor* to “*conditions and stimuli where predictability and controllability are at stake*.” From experiences reviewed in the literature, among others by Mason (2) and Dickerson and Kemeny (7), the Center for Studies on Human Stress of Montreal (Canada) identifies four main stress factors under the acronym N.U.T.S: Novelty, Unpredictability, Threat to the ego and low Sense of control [see also, (9)]. N.U.T.S characteristics appear in the experiences described by the pioneer stress researchers. During and after the introduction of the stress concept, many studies demonstrated its involvement in several psychophysiological disorders (5, 10). Cumulative stressful experiences would be responsible for the allostatic load described by Sterling and Eyer (11) as “*the wear and tear on the body*.”

As far as we understand, each revision of the stress concept aims to reduce the gap between its definition and practical usage. When attempting to evaluate the level of acute stress in a particular individual, a contradictory conclusion is frequently obtained according to whether the stress is assessed on the basis of psychological or physiological observations. This contradiction is particularly more common in the context of sports, where an increase in arousal occurs with, or without N.U.T.S. This discrepancy was also observed in numerous studies outside sports (12, 13). This divergence of conclusions can be explained by a gap between the definition and its application to psychological contexts, as well as by bias related to the utilized stress indicator. A more practical definition of stress would better align the psychological and physiological components. It would also imply a consistent solution to assess the level of acute stress based on physiological indicators.

The impact of stress on health encouraged the health research community to deploy more efforts to better understand the psychophysiological mechanisms involved with stress, including short-term mechanisms for acute stress, and long-term mechanisms involved with chronic stress. Since stress has proven to cause, for healthy subjects, a marked elevation of cortisol levels, most state-of-the-art studies currently use cortisol as a ground truth stress marker. In particular, studies of short-term, *acute* stress widely employ salivary cortisol as a biomarker of stress reactivity (14).The level of salivary cortisol is also frequently employed to assess chronic stress from samples taken at selected days over several weeks or months [see (15)]. Hair cortisol level is also emerging as a valid assessment of cortisol accumulation in the body; this source results from long-term stress (16). For its close relationship with stress and relative ease of sampling, salivary cortisol is currently a leading indicator of physiological stress.

While psychological stress increases cortisol, the reverse cause-and-effect relationship (i.e., that an increase of cortisol involves stress) is not as straightforward, since it depends on the definition of stress. Hellhammer et al. (17) provide biological fundamentals to explain cortisol variations and their relationships with stress, for instance, to avoid a misunderstanding of their relationship that would mislead the conclusions of studies.

Acute stress is commonly measured via an indicator based on the cortisol response magnitude, using a cortisol baseline level at rest. The notion of an *excess* of catabolic mediators, with respect to the actual energetic needs for the task, is generally not addressed. Yet, this notion was introduced by Romero et al. (18), who named it the *homeostatic overload*. For example, a situation that requires a fast energy consumption increase would need sufficient catabolic mediators. For now, such an increase is usually considered stress-related, even though the deployed catabolic mediators fit the energy consumption without significant *excess*.

In long-term studies (15, 19), the circadian cortisol level is considered to assess chronic stress, although without considering the notion of excess. However, by considering this notion, a positive daily stress would mean that the average concentration of catabolic mediators exceeds the quantity required to realize the tasks of the day.

In this paper, we aim to illustrate how the physiological phenomenon of *excess* is related to the psychological components of stress through the N.U.T.S factors, particularly by considering the problem of controlling the maintenance of allostasis, which is defined as *the stability through changes* (11). Based on control theory principles (20), we propose that cortisol variations are part of an anticipative and reactive plan of the brain to fulfill the catabolic needs of current and upcoming tasks.

In summary, our paper contributes by:

- Proposing a framework that describes the components of the control system responsible for maintaining allostasis (as well as their interactions). This framework is based on the allostasis theory proposed by Sterling and Eyer (11), in which the *setpoint* parameters of the body for stability change according to the context and activity. We propose to add some components to their theory. First, the notion of *strategy* is proposed at the basis of setpoint determinations. Second, setpoints are also considered over time in anticipative target trajectories. Finally, regulation is detailed into three asynchronously coordinated regulation loops for the actuators, catabolic mediators, and activators.
- Analyzing the physiological effects of N.U.T.S in the context of maintaining allostasis, and proposing a definition of stress that is closely related to these effects.
- Proposing a consequent, practical solution to measure acute stress.

Our framework is presented in section Framework of Allostasis Control. Section Traditional Indicator: Cortisol Response Magnitude describes previous indicators of acute stress based on cortisol concentration, followed by our proposed indicator. In section Example of Application Using the Physiostress Data Corpus, a first proof-of-concept of this indicator is realized using the open-access data corpus *Physiostress*.

## Framework of Allostasis Control

With the concept of *allostasis*, Sterling and Eyer (11) indicate that the body parameters (such as heart rate, blood pressure, and hormonal levels) required for stability depend on the level of energy that the body has to deploy in its environment [see also (21) for a review of the concept of allostasis]. Each upcoming task involves a specific *setpoint* of the body parameters. The nervous system would be responsible for determining the optimal parameters for body stability at each time (11).

**Figure 1:**
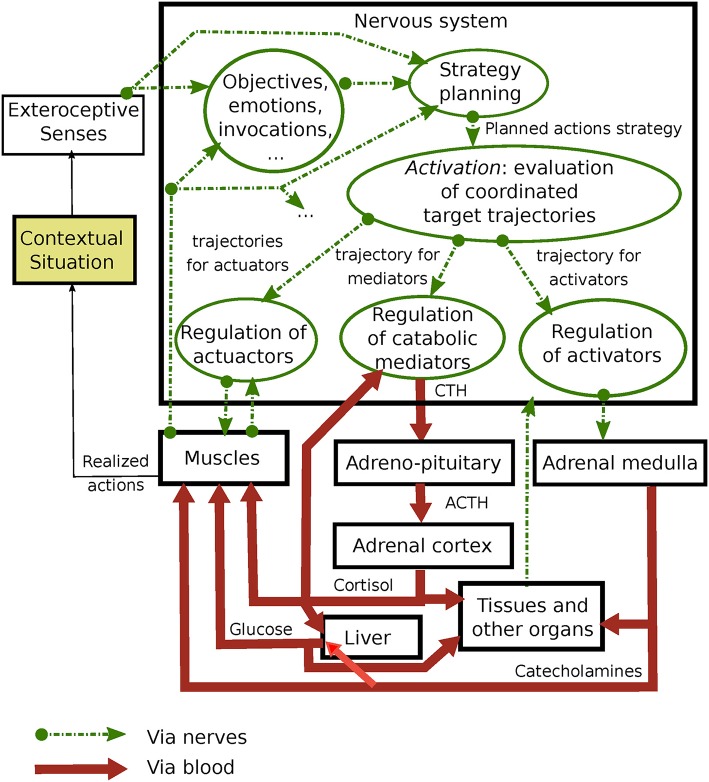
Presentation of the stress response through the proposed framework.

In Figure 1, we propose an overview of different components involved in the problem of maintaining allostasis with their interactions. A contextual situation is perceived by the senses.

A *strategy* refers to an action plan that allows the realization of a particular objective, depending on the perception of the situation, what it invokes, emotions, and internal state variables. A system strategy is broken down hierarchically so as to define the macro actions of the system in its environment. It could include sub-strategies to resolve sub-issues that are part of a global issue and can be related to various duration ranges. While some strategies seem innate, most are built from early experience and life history. For example, if an animal is hidden in a refuge when a predator discovers it, a strategy could be to either flee or freeze (as observed in birds). In order to be effective, this high-level strategy must by supported by coherent control of the muscles and organs by the nervous system. A strategy can thus be implemented through interconnected conscious, subconscious, and autonomic nervous functions (22).

The process of *activation* is related to the application of a specific strategy. It involves coordinating a group of *setpoint trajectories*. We distinguish trajectories that relate to: the desired movements of the actuators (muscles); the targeted deployment of catabolic mediators; and the targeted organs' activation. Hence, our framework is based on the hypothesis that the neural system jointly supervises the activities of the actuators, endocrine system, and adrenal system. Thus, the hypothesis is supported by the synchronicity, namely the consistency, and capacity of activity improvement through the realization of common physical activities. This framework is a logical model of brain functions, implemented through complex neural networks, that is responsible for allostasis control.

In response to a contextual situation, each trajectory of anticipated setpoints feeds a regulation system that aims to maximize the correspondence of the actual body parameters with the setpoint trajectories. Each trajectory is updated every moment according to the current interpretation of the contextual situation and the current states of the internal parameters. The useful anticipation time (thus the trajectory length) varies with the strategy and regulated parameters. For example, it could be ~1 hour for the actuators (in order to plan macro actions) and catabolic mediators (due to the slow regulation loop) while only seconds for the activation.

Catabolic mediators are, essentially, catabolic hormones, and elements that feed body structure activations during an activity, e.g., by enabling energy release and the synthesis of elements for cell survival (23). Catabolic mediators have sensible effects on the operation modes of body structures. In particular, glucocorticoids are transported toward organs via the blood, within which more than 90% of molecules are bound to proteins (24). Bound glucocorticoids (cortisone) are considered *inactive*, while unbound glucocorticoids (hydrocortisone or *cortisol*) are considered *active* since they *activate* cellular processes. The circulating half-life of cortisol varies between 70 and 120 min (25). Since glucocorticoid molecules continuously change from a bound to unbound state (and vice versa) through 11β-hydroxysteroid dehydrogenase activity (25), the blood concentration of unbound molecules stays in equilibrium despite their dissipation through cellular membranes (26), where specific receptors in the cytoplasm trigger domino hormonal reactions. The proportion of bound molecules thus serves as a reserve for upcoming activation needs (27). Catabolic reactions require elements in blood such as glucose, whose production is stimulated (in the liver) by cortisol. Cortisol prepares the body for a sustained activity by stimulating the production of proteins, fat breakdown in most non-nervous tissues, water retention, and enzyme secretion.

The organs' activators are catecholamines (adrenaline and norepinephrine), secreted by the sympathetic nervous system via the adrenal medulla (28). They increase heart rate, blood pressure, breathing, eye pupils, perspiration, and glucose release in the blood. We could consider that catabolic mediators and activators are all involved in the state of *arousal* discussed by Sterling and Eyer (11).

### Strategy Planning

The nervous system develops, through experiences, complex strategies to deal with each type of situation, varying with the hereditary background of the subject, their biological sensitivity to context (29), and their specific history. It jointly develops the ability to evaluate and regulate the catabolic mediators required for these strategies. Korte et al. (30) studied, for example, the evolution of personality types (Hawks–Doves), specifically the strategies for coping with a threat through their physiological and psychological characteristics. Notice that strategy planning applies to animals at different complexity levels [see i.e., (31–33)].

We consider as *successful* a strategy that enables the subject to feel satisfied with his or her performance, and as *unsuccessful* a strategy that leaves the subject feeling unsatisfied. The satisfaction feeling could incorporate complex psychological components up to primitive ones related to survival. The success/failure of the strategies, combined with the amount of stressor exposure and the related experienced emotional load, are important factors for explaining the impact of stressful ordeals on health (21, 34). While the success and failure of strategies are particularly considered in relation to self-esteem, they also play an essential role in subsequent strategy planning. Namely, successful strategies should be reused to efficiently overcome similar situations, while failed strategies should be discarded, which involves the need to process new strategies, with consequent reduced confidence in these untested novel ones. Repeated failure episodes can also cause a lack of self-confidence in the capacity to generate new strategies; thus, there would be a feeling of incapacity to manage the situation. Successful strategies are associated with the concept of eustress and failed strategies with the concept of distress (35, 36).

Now consider the impact of the success or failure of a strategy on the arousal load, defined as the overall arousal level over the duration of an experience. One strategy is more *efficient* than a second if it requires a lower arousal load for an equivalent success. If no successful strategy to deal with a situation is known, the body must be in a high state of alert to dynamically process, evaluate, and adapt new strategies. Conversely, if a successful strategy is known, the neural system would continue to improve its efficiency while adapting it to the particularities of the situation. A greater arousal is therefore expected to respond to unknown situations, modulated by a cost/benefit trade-off aimed at allocating resources according to the importance of the situation (37–39).

The optimization of strategies to deal with a specific situation could converge from one experience to another through a nearly optimal strategy. This convergence should be reflected in the arousal load, such that an optimal trajectory of catabolic mediators is developed behind the optimal strategy. The convergence would mean either an arousal load increase or decrease depending on the energetic demand for the strategy. However, the normal over-activation expected with new, uncontrolled situations, as previously described, implies the typical decrease in catabolic mediators observed in recurrent scenarios (40). This strategy evaluation convergence is considered by Kupriyanov and Zhdanov (36) as a case of eustress and related to a beneficial balance on health. Comparatively, a case of distress would occur with non-convergent strategy evaluations; the result would be a stagnant or elevated arousal load from one experience to another^1^. Distress is considered as the bad form of stress that is related to many health impairments described, for example, in Juster et al. (41). Wüst et al. (42) documented cases of convergent and non-convergent arousal load, without finding reliable explanations for those differences based on purely physiological, and genetic factors. The problems of planning a strategy, with sub-problems of setpoint planning and regulation, may support these observations of decreasing/increasing arousal load.

### Evaluation of Target Trajectories

In control theory, the problem of defining a proper parameter state is isolated from the problem of controlling the system through this reference. In our framework, the setpoint trajectories are the reference of the regulation systems presented in Figure 1, which models the anticipation capability of the neural system. This anticipation capability is observed for the movements, the need in catabolic mediators, and the need in activators.

- When facing a contextual situation, anticipation of movements is a conscious phenomenon.
- Anticipation in catabolic mediators is observed in the typical stress response studied from early experiences (2), where catabolic mediators increase in anticipation of a stressful task. Romero et al. (18) consider the circadian cycle of cortisol as a demonstration of the anticipation capability, which would vary according to the “*the levels needed to respond to predictable environmental changes*” (*Predictive Homeostasis*) and “*the range of the mediator needed to respond to unpredictable or threatening environmental changes*.” We suggest that the large anticipation time observed in catabolic mediator secretion is consistent with the slowness of its regulation loop. Namely, slowly increasing the mediators' concentration is a good strategy to ensure a sufficient concentration level at the time of the activity while facilitating regulation. This type of anticipative closed-loop system is mathematically modeled by Lenbury and Pornsawad (43) through their *feedforward–feedback model* of plasma, adrenocorticotropic hormone (ACTH), and cortisol.
- Anticipation of activation is also observed with a typical increase in heart rate and breath rhythm that precedes a stressful task. The fact that the sympathetic nervous system finely adapts catecholamine secretion to each activity requirement (except in stress scenarios) suggests the presence of a finely supervised control.

### Regulation

Very preliminary attempts to control a system parameter (e.g., a motor speed or position) could be based on open-loop systems, where a specific command is calculated *a priori* for each parameter value and applied during the task regardless of the actual value of the system parameters. These systems are impracticable even in very specific and controlled environments, because the error of the target state, even if small, accumulates rapidly during the task in a butterfly effect. One solution to make the systems functional is to add a sensor to the system that measures the actual state of the parameter (directly or indirectly using knowledge of the relationship between the actual state and the measurements) and to develop *closed-loop control* to remove the error between the actual state and the reference. The success of body regulation systems to converge through well-adapted states suggests the presence of a closed-loop control, which has been especially studied for regulation of actuators and catabolic mediators.

#### Regulation of Actuators

The actuator control system is entirely ensured via nerves from command to feedback [see (44)], a design that enables a fast control loop. Disturbances of this system could be considered in stress evaluation, e.g., based on altered motor patterns (45).

#### Regulation of Activators

Regulation of activators involves two problems: (1) adapting the concentration of catecholamines for activating the organs in a manner to sustain the catabolic activities required for the task, and (2) finely control secretion of catecholamines in levels which are consequent to (1). These two problems responsible for the catecholamine concentration must have distinctive interconnected control loops.

1. To the best of our knowledge, literature does not identify any hormone sensed by the brain clearly involved in a closed-loop regulation of the organs through catecholamine concentrations. Variations in organ activities are detected by nerves, but the role of these inputs in regulation of organs, which may be part of complex neurological pathways, has still to be investigated. A closed-loop regulation system for this system is not biologically assessed. Besides, the hypothesis of an open-loop system does not explain by which mechanisms the sympathetic nervous system may finely adapt, even in anticipation, the organs activities (through secretion of catecholamines) to each activity requirement.
2. Starke et al. (46) propose that *presynaptic autoreceptors* of sympathetic neurons may contribute to a closed-loop regulation of the neurotransmitter release.

The rapidity of the organ responses to catecholamines allows a fine synchronization of their activation to the needs of the actuators. The short half-life of catecholamines (under 2 min) limits their impact on the arousal load in comparison to catabolic mediators such as cortisol.

#### Regulation of Catabolic Mediators

Regulation of the catabolic mediators is performed via the Hypothalamic-Pituitary-Adrenal (HPA) axis in a much slower regulation loop than for control of actuators and activators, due to the blood transmission, cascade of intermediate reactions in the loop, and hormone half-lives. Secreted by the paraventricular nuclei of the hypothalamus, the neurotransmitter corticotropin releasing hormone (CRH) activates adrenocorticotropin secretion by the pituitary gland, which in turn activates glucocorticoid secretion (cortisol and cortisone) by the adrenal cortex (47). The glucocorticoid level elevations may be observed in saliva, plasma and blood serum. By its physical properties (size and viscosity), glucocorticoids reach the mineralocorticoid receptor (MR) and glucocorticoid receptor (GR) in the hippocampus and thus serve as feedback mediators that allow closed-loop regulation of the HPA axis (48, 49).

### Security and Conservation of Resources

Security and conservation of resources are important trade-off criteria (37–39) in the allostasis system. At the level of strategy planning, security favors strategies with a high probability of success, while conservation favors strategies that involve less energy. At the level of setpoint trajectories, security may involve accurate and/or fast movements of the actuators. It would also involve a greater reserve of catabolic mediators in order to feed a fast strategy change due to an unexpected situation turn. Conservation of resources may involve less accurate and/or slower actuator movements. It also requires that the deployed catabolic mediators fit the energy needed to perform the strategy with a minimum of excess (i.e., resulting from the reserve for safety). Finally, the activator concentrations must stimulate the organs according to the needs of the body structures, e.g., in terms of energy, oxygen, and nutriments. Organ over-activation may compromise system integrity to produce useless resources.

**Figure 2:**
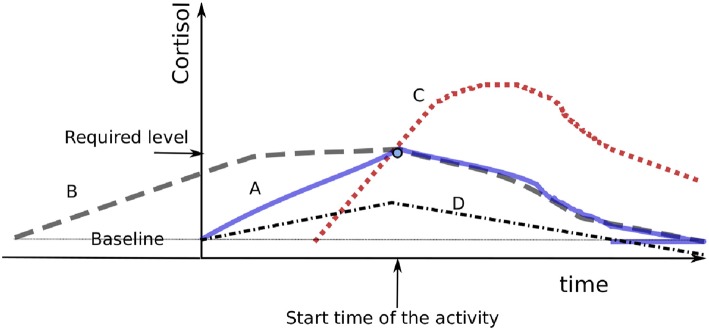
Presentation of four cortisol responses. Curve B presents a too-early response, while curve C presents a late response and curve D presents an insufficient response. Cases B and C increase the area under the curve compared to curve A, which actually presents the best response among the four.

Minimizing the catabolic mediators, and thus reducing the arousal load, is performed when evaluating target trajectories with regards to regulation capability. Figure 2 compares four possible cortisol trajectories. In curve A, the cortisol level gradually increases until it reaches the required level at the time of the activity, then decreases during task realization to the baseline level. Curve B shows a response in which the required level is reached too soon. Curve C shows a late response that requires fast augmentation of the cortisol concentration, which would cause overshooting (named *homeostatic overload* by 40) due to control loop delay. Curves B and C increase, compared to curve A, the arousal load with useless mediators. In curve D, the mediators required to realize the strategy are not reached, a phenomenon that would impair the completion of the strategy and/or reduce the hormonal level below its baseline and compromise vital functions (termed *homeostatic failure* by 40). A hormonal secretion well-adapted to a strategy is sufficiently high to enable the body to efficiently perform the task and acceptably low to be mostly consumed after the task, thus without significant excess.

### Stress and Control Issues

As addressed in the introduction, the contemporary concept of stress tends to be restricted to situations that impact the body's health, as observed with the interpretation of every-day situations as novel, unpredictable, threatening to the ego, and that decrease the sense of control. These situations could, in fact, be viewed as related to one or many issues in the body's response in (i) strategy planning, (ii) targeting setpoint trajectories, and/or (iii) regulation.

#### Novelty

Novelty causes problems in strategy planning (i) and targeting setpoint trajectories (ii). A novel situation involves researching, developing, and testing novel strategies of uncertain energy consumption levels. Consequently, the anticipated setpoints may not accurately fit the effective resources required for task realization. In practice, we would rather talk of degree of novelty. For maximizing security, the brain would evaluate the required catabolic mediators based on the most demanding experiences similar to the current situation. This task requires the brain to evaluate a set of degrees of proximity between the current situation and previous ones. An original situation is subject to being fairly equidistant to many heterogeneous experiences, which must increase the probability of a high reference level. An original situation requires more anticipation time to assimilate the information, plan proper strategies, and activate the body.

#### Unpredictability

Unpredictability involves the brain being surprised by one or many subsequent imminent situations, with the risk of not having time to perform effectively each step of the body's response. Unpredictability then issues problems in (i), (ii), and (iii). It could provoke a kind of panic scenario in which the strategy selection, targeted catabolic resources, and effective activation must be processed quickly, at the risk of hormonal overproduction, as presented in scenario C of Figure 2. If the brain has time to anticipate a situation with a high level of unpredictability, a more secure strategy is to prepare the body for the worst-case scenario with a high level of catabolic mediators. Very original situations without anticipation time (thus situations that involve novelty and unpredictability) provoke a kind of *freeze* reaction, as commonly observed with an animal that crosses a street when a car is approaching. This freeze response could result from a *freezing* strategy (50) as well as from a blockage in strategy planning, in which case other steps are plausibly not performed in time.

#### Threat to the Ego

If the brain strives to maximize security and self-being while avoiding long-term resource waste, protecting self-esteem is of main importance. Basically, a low self-esteem, in addition to a lack of self-love, involves a disagreement with our behavioral modes, thus the need to change them and a requirement for novel strategies to cope with our environment. This lack of confidence in our normal strategy planning mechanisms imperatively compromises our sense of control, discussed in the next point. A threat to the ego emphasizes the global consequences of a failure to control the threat and thus justifies spending more resources on beating it, with more demanding strategies, and/or a larger reserve of catabolic mediators allowing an efficient reaction to unpredictable events.

#### Sense of Control

Lack of control means that the brain knows no efficient strategy to effectively control the situation. The brain is therefore in a search mode where it continuously develops and tests new strategies. Since the strategies are *a priori* undefined or subject to change in progress, the required catabolic mediator levels are accordingly difficult to optimize and subject to being either preventively high or unstable over time.

In light of the consequences of the N.U.T.S situation, we can consider stress as *a state of difficulty in maintaining allostasis*, which typically results in a prolonged state of tension from the consequent excess of catabolic mediators.

With this view, the form of stress may be differentiated into interrelated categories, namely strategic (trouble in performing strategy planning), activation (difficulty evaluating target body activation), or regulation (problems with hormonal regulation). Strategic stress thus involves issues in evaluating a proper strategic response to a situation, i.e., due to novelty or unsuccessful experiences. Activation stress involves trouble in evaluating proper setpoint trajectories for strategy achievement. Regulation stress involves issues in controlling the system structures according to the setpoint trajectories. The intensity of each form of stress has complex, situation-specific relationships with one other. For example, in the case of a fast and complex incoming threat, a high strategic stress level is expected, without there necessarily being significant activation and regulation stress if the importance of the threat is low-ranked (thus not justifying a significant body activation). In the case of a predictable sport (e.g., running in a calm environment) performed by a novice, a high level of activation stress is expected without important strategic stress. The strategy of running, with its sub-strategies that decompose body movements, are indeed usually implicit. A task that must be performed sooner than expected can generate high activation and regulation stress levels with possible low strategic stress. Biological impairments in the HPA axis could firstly generate regulation stress. Over repeated experiences, it should follow activation stress (since anticipation is based on the regulation capability). Biological impairments could also generate strategic stress as improper levels of arousal affect the effectiveness (and even the success) of strategies.

Strategic stress involves mainly conscious mechanisms (with some subconscious mechanisms), since strategies are linked with concrete action plans to deal with the situation. Hence, we can expect it to be more related to the stress felt by the subject rather than other subconscious forms of stress, although they could also be involved in the feeling of being able to handle the threat. However, only activation and regulation stresses are responsible for the cortisol response magnitude, as well as the cortisol excess considered in the evaluation of stress indexes based on salivary cortisol. The possible discrepancy between the intensity of strategic stress vs. activation and regulation stresses could thus contribute to explain the typical low correlation between the stress felt and the ground truth of physiological data. For the rest of the paper, we will use the term stress alone as inclusive of all forms without differentiating them.

## Traditional Indicator: Cortisol Response Magnitude

The cortisol response magnitude is currently a gold standard indicator for stress assessment. This indicator assumes that the cortisol increase during an experience is directly related to the stress level. It can be computed using different methods, e.g., based on the *maximum cortisol increase* (*MCI*) or the global cortisol increase using the *area under the curve* (*AUC*) of the cortisol response.

*MCI* is typically evaluated with the increase of cortisol following the task, i.e., the difference between the maximum cortisol value after the beginning of the task up to the end of the recovery period and its minimum value before the task beginning. This increase can be normalized based on the minimum (or average) cortisol values during the period of rest (anticipative and recovery periods):

(1) $$C_{rep}=\frac{C_{max,post}-C_{min,pre}}{0.5*\left(C_{min,pre}+C_{min,post}\right)}$$

where “pre” refers to the period before the stressful experience and “post” the period after.

AUC can be evaluated by integrating the cortisol response parts. By linking the cortisol point with straight lines, we obtain the integral by summing a set of trapezoidal areas, as detailed by Pruessner et al. (51). These authors distinguish the AUC with respect to ground (AUC_C_) from the area under the curve with respect to increase (AUC_I_). The authors assert that the second quantity, theoretically more related to acute stress, is sensitive to the error on the cortisol sample that precedes the stress test (since the increase is relative to its value).

The assumption that the cortisol response magnitude indicates the intensity of acute stress could be unrealistic for the following reasons:

- *Problems of underestimation*. In the first rest period, it is assumed that the participant is not stressed. This assumption could be unrealistic if the first period of rest follows a stressful task or if the participant anticipates the stressful task of the following period. A consequent high cortisol level during the period of rest causes an underestimation of the cortisol response.
- *Problems of overestimation*. The maximum cortisol increase does not account for the catabolic needs of the upcoming task. In our stress definition, the stress concept is linked only with the excess of catabolic mediators and not with the part effectively consumed to perform the task, which may result from an efficient allostasis control. The consumed part of catabolic mediators overstates the intensity of acute stress.

### Proposed Indicator: Excess Cortisol Secretion

In section Stress and Control Issues, we discussed that issues in processing the body's response, especially observed in N.U.T.S situations, typically provoke catabolic mediator overproduction. Catabolic hormone excesses, compared to the quantity required to perform the task, then appear as a relevant stress indicator.

**Figure 3:**
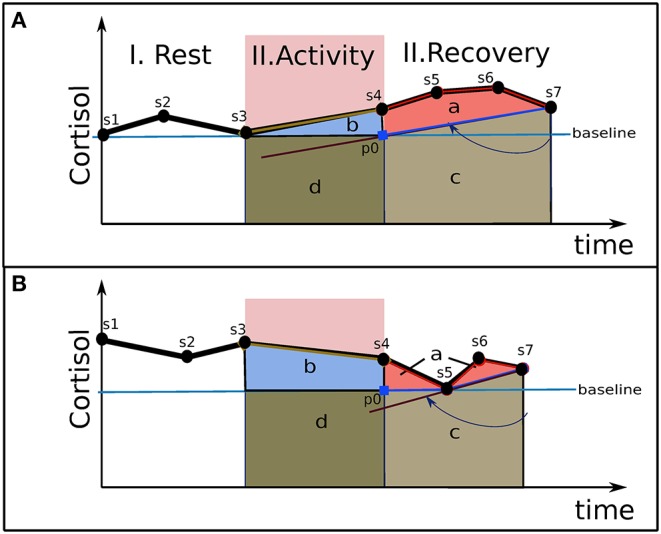
Two examples of the determination of excess cortisol load. Points [s1, s7] are the concentrations of the cortisol samples. Four areas under the curve are presented: (a) Excess of cortisol load; (b) cortisol load for the activity; (c) basal and anticipative cortisol load of the recovery phase; (d) basal cortisol load for the activity. **(A)** Experience 1; **(B)** Experience 2.

Figure 3 presents two examples of cortisol responses from which we strive to identify an area related to cortisol secretion excess. It represents a typical experience for studying acute stress, which involves rest, task, and recovery periods. Salivary cortisol is sampling during the experience about each 15-min. The two experiences in Figure 3 include, for example, seven samples [s1, s7]. Basal cortisol, represented by a straight horizontal line, crosses the minimum value (see s3 in A, s5 in B) of the cortisol response over the experience. Each area under the cortisol response represents a *cortisol load*. The area *b*, delimited by the baseline and the curve of cortisol concentration, represents the cortisol load consumed for the activity plus an excess secretion. Due to the elimination time of cortisol (see section Framework of Allostasis Control), this excess must still be observed in the recovery period. Moreover, due to the slowness of disengagement with the task combined with the slowness of the hormonal regulation (as discussed in section Regulation), a potential prolonged over-secretion may continue during the recovery period. Consequently, the recovery period will allow estimation of the total excess secretion related to the experience, related to area *a*. Area *c* is not considered in the excess part, since it may be related to a preparation for activities expected after the recovery period. In order to evaluate area *c*, we spin clockwise a straight line around the last cortisol point (see s7) until it reaches either the baseline at the beginning of the recovery period (see p0) or a previous cortisol point during the recovery period (see s4–s6). Area *c* is delimited in each point with the higher limit between this straight line (which crosses p0-s7 in A and s5-s7 in B) and the cortisol baseline.

The proposed stress indicator is the *excess proportion of cortisol load* (EPCL), which is the proportion between the excess cortisol load (area *a*) and the cortisol load related to the activity (area *a* and area *b*):

(2) $$\beta =\frac{area(a)}{area(a)+area(b)}.$$

This indicator level denotes how much, on a 0–1 scale, the cortisol load generated for the task exceeds its needs. EPCL then scales the *state of difficulty in maintaining allostasis* (namely the stress definition of section Stress and Control Issues), in opposition to traditional indicators based on the cortisol response magnitude which implicitly relate a catabolism increase to *stress*. Hence, it would bring a measure more in phase with the established psychological and physiological stress components. In Equation (2), the excess part is scaled with the needs of the individual for the specific task, represented in area (b), which enables the use of EPCL to compare the *stress* response of individuals who have different catabolic needs for achieving the task.

## Example of Application Using the Physiostress Data Corpus

### The Physiostress Corpus

Our data corpus presented in Boucher et al. (52) is considered in this section to provide a practical example of stress levels obtained with the proposed indicator of acute stress EPCL^2^, and to compare it with previous methods based on the cortisol response magnitude. In particular, we consider data of 25 participants who performed a 2-days experimental procedure. The first day was dedicated to a stress task and the second day to a social task followed by a physical activity task. Each task period was surrounded by a rest and recovery period.

During both days:

- The participants' salivary cortisol was sampled throughout the experiment approximately every 15 min and again during transitions between the tasks.
- The participants indicated their level of perceived stress (no, low, average, or high) every 2 min during the rest and the recovery periods and at the beginning, middle, and end of each task. The stress of the participants was thus considered for both days.

#### Day 1: Trier Social Test Task (TSST)

The TSST is a recognized test for inducing moderate acute stress in a majority of participants (53). The experience includes the following steps:

1. The participant has a rest period of 15 min;
2. The participant is advised to prepare, for 10 min, a 5-min oral presentation to convince, in a second room, three evaluators (two in our version) that he, or she is the best candidate for a monitor job at a summer camp with children.
3. The participant goes into the second room and performs his or her presentation for 5 min. The evaluators attempt to display a neutral attitude without emotional expression. After the presentation, the evaluators ask the participant to perform an arithmetical task as fast as they can.
4. The participant returns to the first room for a 45-min rest period.

#### Day 2: Social Task and Physical Activity

On the second day, the stress task is replaced by a short conversation with the instructor, who does their best to keep the conversation pleasant and relaxed for the participant. The task preparation in the first room is replaced with an additional 10 min of rest. After the social task, the instructor guides the participant through a 10-min series of 30-s exercises (standstill running and squats) split by 10 s of rest in order to provoke a physiological activation.

## Results

**Figure 4:**
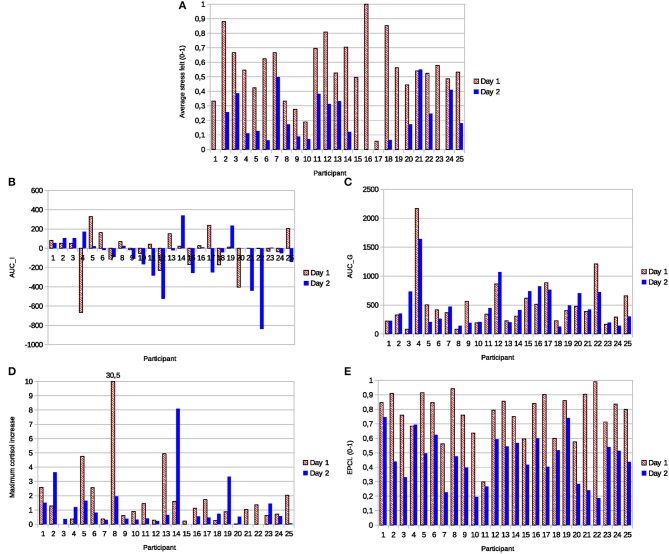
**(A)** Stress felt in relation to different cortisol-based indicators of stress, namely **(B)** the area under the curve with respect to increase (AUC_I_), **(C)** the area under the curve with respect to ground (AUC_C_), **(D)** the maximum cortisol increase, and **(E)** the excess proportion of cortisol load (EPCL).

Based on salivary cortisol levels in the Physiostress corpus, we illustrate the problems of underestimation and overestimation presented in section Traditional Indicator: Cortisol Response Magnitude. These problems could explain many unrealistic stress evaluations based on the response magnitude, namely numerous low cortisol magnitude responses observed in our corpus for day 1, and many high cortisol magnitude responses for day 2 because of the physical activity. Indeed, Figure 4D presents classical metrics used by researchers, including the MCI, AUC_G_, and AUC_I_, in comparison with our proposed measure (EPCL), and the average stress felt by each participant in both situations.

Eight out of 25 participants (27%) had a lower maximum cortisol increase on day 1 of the TSST (stress task) than on day 2 with the social talk and sporting activity (Panel D). In panels (B) and (C), 8 and 15 participants had lower AUC_I_ and AUC_G_ on day 1, respectively. AUC_I_ was even negative for 10 experiences on day 1. Besides, all participants except one reported higher average stress felt during the task on day 1, as expected by the nature of the tasks (Panel A). Therefore, we could consider either that many participants overestimated their stress for day 1, or that the cortisol response magnitude is not a realistic indicator of the stress felt by those participants, plausibly because of the problems of underestimation and overestimation presented in section Traditional Indicator: Cortisol Response Magnitude.

Panel (E) of Figure 4 shows the EPCL for the same participants. The first day of TSST induced, for all but one participant, a higher EPCL than the second day with the social and sportive tasks. Even though this participant was not the one who reported lower stress on day 1, these results are, on the whole, more coherent with the higher stress level expected from the TSST and the self-reported stress of the participants. They also suggest that EPCL would hardly be biased by the physical activity linked with the task.

In panel (A), the average stress felt dropped 36.8% from day 1–2, while the average EPCL decreased by 30.8%. Indeed, the experience of the social conversation and physical activity of day 2 did not generate important strategic stress, data that would explain why participants reported lower stress levels for this day. The correlation coefficient between the stress felt and the EPCL was 0.305 (against −0.05 for MCI, 0.07 for AUG_G_, and −0.32 for AUC_I_), which appears coherent with the low correlation expected between the intensity of the strategic stress and the intensity of the activation and regulation stresses (related to the EPCL).

Each stress index produced values that were slightly correlated with those of other indexes (EPCL/MIC: 0.31, EPCL/AUC_G_: 0.07, EPCL/AUC_I_: 0.46, MCI/AUC_G_: −0.18, MCI/AUC_I_: 0.29, AUC_G_/AUC_I_: −0.32). These correlations mean that the relative stress from day 1–2, and from one participant to another, varied in a specific manner, often contradictive, for each index.

## Conclusion

In this paper, we propose a framework for depicting the main components of allostasis control, which is deployed on three levels for strategy planning, evaluation of target trajectories, and regulation. In order to map physiological and psychological stress components into a same biological phenomenon, we define *stress* as *a state of difficulty in maintaining allostasis*, which could result either in *strategic, activation*, and/or *regulation stresses*, depending on which level is affected. This definition contrasts with previous ones that implicitly relate a catabolism increase to stress, which assumption causes misalignment between the psychological and physiological stress components as well as between the health effects of *stress*. By contributing to reduce discrepancy between the situations related to stress, this definition should contribute to improve the practical meaning of the term. It also opens research to analyze each form of stress individually, so as to better understand their respective properties, causes and impacts, without confounding them under the same term (which may indeed cause contradictive conclusions if the properties, causes, and impacts vary with the stress form). For example, further works could investigate the neurological properties of *strategic stress* and their involvement in the development of neurological disorders.

Activation and regulation stress commonly cause an oversecretion of catabolic mediators, including cortisol. Consequently, the EPCL is proposed as an indicator of stress. Experiments in section Example of Application Using the Physiostress Data Corpus demonstrate the potential of this stress indicator, which offers, with our data corpus, stress levels that are more consistent with the psychological components of stress, and less sensitive to physical activity. These results also present drastic variations of stress assessment of a participant from an indicator to another, which suggests that the choice of the stress indicator may impact the conclusion of a study. The EPCL applicability to long-term and chronic stress should be investigated in further works.

In our view, the quality of each stress indicator must be addressed based on their theoretical fundamentals. The stress indicators, with the stress concept itself, have in all likelihood evolved together so as to become more and more informative on the health state of the subject and their predisposition to develop health problems. We believe that the proposed theoretical framework provides a step in this direction by clarifying the nature of stress in the problem of maintaining allostasis.

## Data Availability Statement

The datasets generated for this study are available on request to the corresponding author.

## Ethics Statement

The protocol for generating the Physiostress data corpus was approved by the ethical committee of the CIUSSS of the East-Island of Montreal (project 2015–218).

## Author Contributions

PB developed the theory and the proposed the method with the support of PP, who contributed to important aspects during the process to ensure the quality of the content and the form of the article.

### Conflict of Interest

The authors declare that the research was conducted in the absence of any commercial or financial relationships that could be construed as a potential conflict of interest.

## Footnotes

^1^Note, however, that eustress, with convergent strategies, would imply an increasing arousal load in a case where it is insufficient for the task during the first experiment, e.g., due to a lack of anticipation and/or engagement.

^2^Notice that EPCL is computed as described in section Proposed Indicator: Excess Cortisol Secretion, and that results are first time published in section Day 2: Social Task and Physical Activity.
